# Does Growing up in Urban Compared to Rural Areas Shape Primary Emotional Traits?

**DOI:** 10.3390/bs7030060

**Published:** 2017-08-29

**Authors:** Cornelia Sindermann, Keith M. Kendrick, Benjamin Becker, Mei Li, Shijia Li, Christian Montag

**Affiliations:** 1 Department of Molecular Psychology, Institute for Psychology and Education, Ulm University, 89081 Ulm, Germany; 2 Key Laboratory for NeuroInformation/Center for Information in Medicine, School of Life Science and Technology, University of Electronic Science and Technology of China, Chengdu 611731, China; k.kendrick.uestc@gmail.com (K.M.K.); ben_becker@uestc.edu.cn (B.B.); 3 Student Counseling Center, Beijing University of Civil Engineering and Architecture, Beijing 100044, China; limei@bucea.edu.cn; 4 School of Psychology and Cognitive Science, East China Normal University, Shanghai 200062, China; sjli@psy.ecnu.edu.cn

**Keywords:** childhood, (mega-)cities, primary emotional traits, urbanicity index, Germany, China

## Abstract

Growing up in urban areas represents a possible risk factor in the genesis of psychopathologies. The aim of the present study was to investigate the link between urbanicity variables and indicators for psychiatric disorders. We investigated a potential association between primary emotional traits and urbanicity variables in 324 individuals from Germany and 713 individuals from China. Higher scores in the urbanicity index in childhood were inversely associated with FEAR and SADNESS only in adult Chinese females. These effects seemed to be driven by living in Chinese mega-cities, because a parallel sample from Germany and China (contrasting upbringing in cities with the categories <10,000 inhabitants, ≥10,000 inhabitants (but <100,000), and ≥100,000 inhabitants) resulted in weaker, but more similar effects in females in both countries. Additional associations could be observed with higher PLAY and urban upbringing in Chinese males. The results seem surprising, given an expectation of adverse emotional effects from growing up in todays’ mega-cities compared to rural areas. Although we do not want to over-interpret our findings (given rather small correlations and multiple testing issues), they should encourage researchers to consider including urbanicity variables in personality neuroscience and personality oriented clinical psychiatric research.

## 1. Introduction

According to worldometers.info, 7.4 billion humans live on Earth as of January 2017 [1]. Although living in the city has many advantages such as good access to medical treatment, higher number of jobs, higher salaries, and also richer cultural life, several studies indicated that living in cities causes stress via noise, pollution, and hectic living style (e.g., [2,3]). In addition, factors such as differences in social support (from family and/or friends), stressful life events and familial liability between urban and rural areas could also impact the quality of life [3,4,5,6,7]. Moreover, urban versus rural environments differ in their natural resources with urban areas usually providing less green restorative areas, which have been shown to be of relevance to foster well-being and health [8,9]. Clearly, urban areas rather provide the opposite of natural environments, namely so called “built environments”. Broadly, a “built environment” describes spaces, products, and buildings that are made by humans. These cannot only impact physical but also social environments (as mentioned above via noise and pollution; to name just a few). This in turn can also influence (mental-)health [10]. The link between natural environments and well-being has also been put forward by a study demonstrating that a walk through the city forest compared to a walk through “grey building areas” has a positive effect on both cognitive functions and mood elevations [8]. This underlines the importance to implement green spaces into today’s mega-cities. Taken together, living in (mega-)cities might be a causal factor for the development of psychiatric disorders [11]. Accordingly, it has been demonstrated that the prevalence of schizophrenia and depression is higher in urban-living as opposed to rural-living populations (e.g., [12,13,14,15]).

One prominent neuroscientific approach to understand the development of psychopathologies represents Panksepp’s affective neuroscience (AN) theory [16,17]. Focusing on electrical brain stimulation he mapped neural circuitries underlying the seven primary emotional systems (PES) called SEEKING, LUST, CARE, and PLAY (positive emotions); and FEAR, ANGER, and SADNESS (negative emotions) driving the human psyche/behavior in a bottom-up fashion. These systems reflect in-built tools for survival which are highly evolutionarily conserved across the mammalian brain (for further information on these PES and its evolutionary functions see Table 1). Imbalances in these different systems are associated with psychopathologies [18], e.g., higher FEAR/SADNESS together with lower SEEKING activity represents the state of depression [19]. Moreover, high SEEKING could be among the foundations of schizophrenia [18]. The neural circuitries underlying these PES are influenced by genetics and environment [20,21]. Moreover, it is well-known that the development of these PES is particularly vulnerable to impairment during early life [22]. Thus, even if the PES are (strongly) influenced by genetics, environmental factors as well as gene by environment interactions—especially during early life—can also shape PES in different directions. Finally, individual differences in PES likely represent the phylogenetically oldest basis of human personality, driving behavior in a bottom-up fashion (from ancient subcortical regions). In a recent cross-cultural study by Montag and Panksepp, it has been demonstrated empirically that high SEEKING could indeed form the basis of Openness to Experience, high PLAY the basis of Extraversion, high CARE/low ANGER the basis of Agreeableness and high FEAR, SADNESS, ANGER the basis of Neuroticism [23]. Therefore, the investigation of individual differences in PES in the context of urbanicity variables is of high interest not only in research about the etiology of psychopathologies but also to gain putative insights into personality development.

With the present (exploratory) research, we aimed to investigate if upbringing as well as current living in small or large cities in Germany and China is associated with a different set of primary emotional traits (PETS) in adults. Hence, we have focused on environmental influences on personality development (as individual differences in these PETS potentially build the oldest evolutionary basis of human personality) [21,23,24]. Based on the cited previous findings, we expected AN’s positive emotional traits to be inversely linked with urbanicity during childhood and current living in a city, whereas negative emotional traits should be positively linked.

## 2. Materials and Methods

### 2.1. Sample

Data were collected at universities in Germany (Ulm) and China (Beijing, Chengdu, Shanghai). Thus, most of the participants were students. Due to the similar cultural backgrounds of the participants, the three samples from China were combined. Complete data were obtained from *n* = 324 participants in Germany (111 males, 213 females; age: M = 24.63, SD = 9.37) and *n* = 713 participants in China (434 males, 279 females; age: M = 20.56, SD = 2.51). All subjects gave their informed consent for inclusion before they participated in the study. The study was conducted in accordance with the Declaration of Helsinki, and the protocol was approved by the local ethics committee at Ulm University, Ulm, Germany and by the local ethics committee at UESTC in Chengdu, China.

### 2.2. Questionnaires

All participants filled in the Affective Neuroscience Personality Scales (ANPS), a self-report measure constructed on the background of empirical evidence from AN theory [25,26]. Note that, in the German part of the study, the participants filled in the 110-item version of the ANPS and in the Chinese part of the study the slightly revised version consisting of 112 items [25,26,27]. The Chinese version of the ANPS has been translated in two steps by the present work group. First, the questionnaire was translated from English into Chinese and then secondly it was independently re-translated back into English. The English retranslation and the original version were checked for comparability to make sure that the original meaning of the items of the ANPS did not change. This Chinese version will be published shortly elsewhere (including detailed information about the translation process and using different data). Each PET scale of the ANPS consists of 14 items other than the Spirituality scale which consists of 12 items. All items are answered using a four-point Likert scale ranging from “1 = strongly disagree” to “4 = strongly agree”. The questionnaire assesses the PETS “SEEKING, FEAR, CARE, ANGER, PLAY, and SADNESS” as well as an additional scale for Spirituality. Spirituality does not represent a primary emotional trait, but is included due to its potential effects in the treatment of patients afflicted with psychopathologies (other personality theories also include such a dimension: self-transcendence in Cloninger’s biosocial theory of personality [28]). Items for LUST have not been constructed due to possible negative carry over effects in answering the other scales of the ANPS. Such negative effects could result from biased tendencies in answering items linked to one’s own sexual preferences, behavior, and overall sexuality, which could then also affect the remaining items of the ANPS. The internal consistencies for the different scales are presented in Table 2. 

Additionally, an urbanicity index questionnaire inspired by Lederbogen et al. and Mortensen et al. was administered [3,29]. In Germany, participants were asked how long (in years) they have been living in a city with <10,000 inhabitants (answer-option 1); ≥10,000 inhabitants (but <100,000), (answer-option 2); or ≥100,000 inhabitants (answer-option 3) up until the age of 15 years. In China, participants were asked the same question but different categories of cities were added as answer options to account for the high number of inhabitants in many Chinese cities. Thus, they were asked how long (in years) they have been living in a city with <10,000 inhabitants (answer-option 1); ≥10,000 inhabitants (but <100,000), (answer-option 2); ≥100,000 inhabitants (but <1 million), (answer-option 3), ≥1 million inhabitants (but <5 million), (answer-option 4); ≥5 million inhabitants (answer-option 5) up until the age of 15 years. The years lived in the respective kind of city were then multiplied with the respective size factor ((1) for “<10,000”, (2) for “≥10,000; <100,000”, ..., (5) for “≥5 million”) and summed to create one urbanicity index, which could range from 15 to 45 in the German sample and from 15 to 75 in the Chinese sample (as a manipulation check, we made sure that all included participants reached a sum of 15 years; see formulas for calculating the urbanicity index below). Higher scores indicate a greater number of years spent in more urban areas as a child/adolescent. Moreover, the participants were asked to give information about the number of inhabitants in their birth-town and their current residence (an ordinal variable where participants could choose between options ranging from (1) to (5) based on the categories mentioned above).

Formula for calculating the urbanicity index in the German sample:

(N_years(<10,000)_ × 1) + (N_years(≥10,000; <100,000)_ × 2) + (N_years(≥100,000)_ × 3)

Formula for calculating the urbanicity index in the Chinese sample:

(N_years(<10,000)_ × 1) + (N_years(≥10,000; <100,000)_ × 2) + (N_years(≥100,000; <1,000,000)_ × 3) + (N_years(≥1,000,000; <5,000,000)_ × 4) + (N_years(≥5,000,000)_ × 5)

### 2.3. Statistical Analyses

All analyses were implemented independently in the samples from Germany and China. First, associations between age and the ANPS/urbanicity index were calculated using Pearson correlations. In addition, a MANOVA was implemented with gender as an independent variable and the ANPS/urbanicity index as dependent variables to check for gender effects. Next partial correlations between the urbanicity index and the ANPS were calculated controlling for age. All correlations are presented for the whole samples as well as separately for males and females (see Section 3.4). To obtain more robust estimators of the correlations, we also calculated confidence intervals (using bootstrapping methods) for all correlations. As we found some descriptive differences in the correlations between males and females, we also investigated the significance of the gender differences in these correlations by further conducting Fisher’s z-tests (CorrComparer V1.0). Additionally, we implemented regression analyses separately in the samples from Germany and China. The effects of gender, urbanicity index, and the interaction term (all included in one step) were investigated on the respective ANPS scales, in which we found (descriptive) differences in the correlations between males and females as depicted in Table 4.

For analyzing the effect of the birth-town or current residence, separate MANCOVAS were calculated, with age as a covariate and separately for German and Chinese subjects (in each case for males and females combined as well as separately).

## 3. Results

### 3.1. Descriptive Statistics

The descriptive statistics for all variables of interest in both samples from Germany and China are presented in Table 3. Note, that age and gender variables for each sample have been presented in the Section 2.1.

### 3.2. Effects of Age and Gender

In the German sample, age correlated significantly with FEAR (r = −0.18, *p* = 0.001), ANGER (r = −0.11, *p* = 0.049), PLAY (r = −0.26, *p* < 0.001), SADNESS (r = −0.14, *p* = 0.009) and the urbanicity index (r = 0.15, *p* = 0.008). In the Chinese sample, age correlated significantly with the ANPS scales CARE (r = −0.12, *p* = 0.002), PLAY (r = −0.09, *p* = 0.020), SADNESS (r = −0.19, *p* < 0.001), and Spirituality (r = −0.09, *p* = 0.015); as well as the urbanicity index (r= −0.25, *p* < 0.001).

Significant gender differences were also found: In the German sample females scored higher than males in FEAR (F(1, 322) = 21.83, *p* < 0.001), CARE (F(1, 322) = 45.81, *p* < 0.001) and SADNESS (F(1, 322) = 24.77, *p* < 0.001). In the Chinese sample females scored significantly higher in FEAR (F(1, 711) = 16.79, *p* < 0.001), SADNESS (F(1, 711) = 40.97, *p* < 0.001), and the urbanicity index (F(1, 711) = 8.01, *p* = 0.005); whereas males scored significantly higher in PLAY (F(1, 711) = 5.27, *p* = 0.022).

### 3.3. Correlations between Urbanicity Index and Size of Birth-Town

As a manipulation check, we computed the correlation between the urbanicity index and the population size of the birth-town. As expected, the association is highly positive (German sample: r = 0.68, *p* < 0.001; Chinese sample: r = 0.88, *p* < 0.001).

### 3.4. Associations between the ANPS and Urbanicity Variables

As shown in Table 4, no significant correlations between the urbanicity index and any PET dimensions were found in the German sample. In the Chinese sample, a significant positive association was found between the urbanicity index and PLAY in male subjects only (Fishers z-Test revealed no significant difference in the correlations between Chinese males and females: z = 0.92, *p* = 0.18, one tailed test). Additionally, significant negative correlations were found between the urbanicity index and FEAR and SADNESS only in Chinese female subjects (Fisher’s z-tests revealed significant differences in these correlations between Chinese males and females: FEAR: z = 1.71, *p* = 0.04; SADNESS: z = 1.70, *p* = 0.04; one tailed tests). The reported correlations in Table 4 are already controlled for age. Of note, only the significant negative correlation between the urbanicity index and FEAR in Chinese females (*p* = 0.002) would still reach significance after Bonferroni correction for multiple testing (*p* = 0.05/6 tests for 6 PETS = 0.0083).

With respect to the associations between the ANPS scales and birth town/current residency in the German or Chinese sample, one finding turned out to be of interest underlining the correlation between lower FEAR and higher urbanicity index in Table 4 for the female Chinese group. In the Chinese female sample, FEAR did decrease significantly with an increasing number of inhabitants in the town of current residence (F(4, 273) = 6.52, *p* < 0.001). Further associations gained significance, but did not survive correction for multiple testing (none of the corresponding F-tests reached a significance of 0.05/6 tests for 6 PETS = 0.0083 or lower).

### 3.5. Regression Analyses

As seen in Table 5, gender had a significant effect on FEAR with females showing higher scores compared to males in both samples (from Germany and China). Additionally, the interaction term of gender and urbanicity index had a significant effect on FEAR in the Chinese sample with a stronger negative association in females compared to males. Only in the Chinese sample was the urbanicity index positively related to PLAY, but neither significant gender nor significant gender by urbanicity index interaction effects could be observed. Finally, females showed significantly higher scores in SADNESS compared to males in both samples. Additionally, the interaction effect of gender and the urbanicity index on SADNESS was significant in the Chinese sample. Again, a stronger negative association was found in Chinese females compared to males. However, only the significantly higher scores in FEAR and SADNESS in Chinese females (compared to Chinese males) and the positive association between urbanicity index and PLAY in the Chinese sample would survive correction for multiple testing (*p* = 0.05/6 tests for 6 PETS = 0.0083).

### 3.6. Additional Analyses in a “Small” Chinese Sample Adjusted to the Urbanicity Index Categories in the German Sample

We additionally investigated whether growing up in a city with more than 1 million or even more than 5 million inhabitants drives the correlations between the urbanicity index and PETS which were only observed in the Chinese sample. For this purpose, and also for better comparison with the correlations found in the German sample, a subsample of the Chinese data was reinvestigated, including only participants living in category 1–3 cities (but not 4 and 5) until the age of 15 (i.e., in parallel with the German cohort). This “small” sample includes *n* = 290 (*n* = 189 males, *n* = 101 females) participants.

Means and standard deviations of all variables of interest in the “small” Chinese sample as well as correlations between the urbanicity index and PETS are presented in Table 6. Only the positive correlation between the urbanicity index and SEEKING in the female sample reached significance (*p* = 0.036), but would not survive correction for multiple testing (*p* = 0.05/6 tests for 6 PETS = 0.0083). In addition, this association (barely) did not significantly differ between males and females (z = −1.47, *p* = 0.07; one tailed test).

## 4. Discussion

We aimed to establish a link between PETS from Panksepp’s AN theory and urbanicity variables. Such associations would provide some first insights for potential causes underlying the observed higher prevalences of depression/schizophrenia in urban compared to rural areas since variations in PETS have been considered as contributing to these disorders [18]. Moreover, such associations would provide insights into personality development in the context of urbanicity variables, because individual differences in primary emotional systems are likely to represent the phylogenetically oldest parts of human personality.

Two significant and unexpected findings were revealed. In contrast to the literature reporting higher depression rates in individuals living in cities compared to rural areas, Chinese females with a greater duration of upbringing in larger cities showed lower FEAR and SADNESS scores. As high FEAR and SADNESS are hallmarks of depression [19] this seems surprising and demonstrates that many other factors need to be taken into account to understand which contribute most reliably to the association between urbanicity variables and the occurrence of psychopathologies (e.g., gender, education, and age; moreover, variables more directly linked to features of built vs. natural environment should be mentioned (e.g., [8,30])). In addition, Wang suggests the inclusion of “immigration status, race, working status, marital status, and the provinces where they live” (this study was conducted in Canada) [15] (p. 19). Finally, the genetic underpinnings contributing to urbanicity effects should be investigated in the future. As mentioned in the introduction of the present manuscript, genetic underpinnings are of importance in the development of the PES: on the one hand, as independent factors directly influencing the PES, but on the other hand clearly also as resilience/vulnerability factors influencing the development of the PES in early life in interaction with environmental influences such as an urban upbringing. For example, a genetic marker linked to individual differences in SADNESS or FEAR might only then result in an overtly high SADNESS/FEAR system (which makes a person vulnerable towards affective disorders), when adverse environments impact on the developing child/adolescent. This has been often coined as inherited stress sensitivity [31]. In developed cities, more secure environments in terms of jobs provision for parents (hence financial security for family and the offspring) or better health care might buffer the effect of genetic risk variants.

In the present study, it could be hypothesized that being brought up in modern (mega-)cities in China provides individuals with more extensive social networks and greater lifestyle and employment possibilities, which could buffer against high FEAR and SADNESS compared to living in more rural parts of China where social and employment opportunities may be more restricted. This may be particularly the case for females, because—at least in our data sets from Germany and China—females compared to males had significantly higher scores in these domains. This could represent a particular vulnerability towards stress exerted from the environment on females (note that gender differences in negative emotionality occur often, but not always; e.g., see rather modest differences in these traits in Davis et al. [25] (p. 59)). In contrast, we did not observe the same results in Germany, although the associations between FEAR, SADNESS and urbanicity index pointed in the same direction (negative correlations, but were much smaller than in China). Interestingly, when contrasting the female subsample in China living in the smaller urban cities (paralleling the size of German and Chinese cities), the correlations become more similar (FEAR–urbanicity: r = −0.09 (German females) vs. r = −0.13 (Chinese females), SADNESS–urbanicity: r = −0.04 (German females) vs. r = −0.07 (Chinese females), all not significant). Hence, the association between lower SADNESS and FEAR being related to more urban upbringing seems to be driven most by living in mega-cities. Of further note was the finding that, in the “small” Chinese sample, chosen to more directly parallel the German one, a further significant finding appeared with higher SEEKING associated with upbringing in urban areas. Higher SEEKING (e.g., also higher dopamine activity via D2 receptors in the striatum) [17,32] might also be associated with higher positive symptoms in schizophrenia patients [18]. In line with our findings, Tang et al. reported that Chinese female schizophrenia patients tend to have more positive symptoms [33]. This fits with the observation that more individuals suffering from psychotic symptoms/disorders can be found in urban vs. rural sites (e.g., [34]). Nevertheless, we explicitly mention as a limitation that we investigated mostly healthy student samples in Germany and China and do not want to over-interpret our findings in the context of psychopathological disorders. Moreover, the observed correlation is rather small (r = 0.21) and SEEKING as a PET is clearly only one factor among many potentially linked to schizophrenia. 

Finally, a positive correlation only appeared for males between PLAY and urban upbringing in the Chinese sample. Perhaps modern Chinese cities provide males with larger opportunities to nourish their in-built PLAY circuitries (via many entertainment possibilities in adolescence) potentially also leading to more extraverted behavior in their personalities (see [23]). However, it should also be mentioned that PLAY behavior is best nourished by rough and tumble PLAY in early childhood, which is clearly more dependent on play partners and a secure environment than simply on having access to modern forms of entertainment. In terms of the PLAY—extraversion link, one should keep in mind that extraversion consists of many sub-facets including being talkative, socially active, assertive, and less shy/bashful [35]. Playing itself is important for nurturing social competencies [36] and through this also potentially developing greater extraversion (e.g., by playing the leader in the kindergarten group—hence becoming more assertive). Future studies are required to show which facets of extraversion are most strongly linked to the PLAY system as assessed with the ANPS. Moreover, why PLAY in particular is related positively to the urbanicity index in male Chinese needs (a) to be replicated in further studies and (b) explored in more detail by also including further personality measures as well as variables assessing activities being pursued by children/adolescents in today’s mega-cities.

In general, our findings need to be considered as preliminary given issues of multiple testing and the rather small correlation coefficients observed. After correction for multiple testing only the association between FEAR and the urbanicity index in Chinese females would survive (correcting the alpha of 0.05 by six tests for six PETS). A further limitation of the present study is its cross-sectional character with participants self-reporting the number of years of upbringing in different cities. Although the variables urbanicity index (upbringing; past childhood) and PETS (assessed in the present) have a clear sequential timeline, one should be cautious to infer causality. Moreover, it is questionable if our findings can be transferred to other countries or to generations growing up today, because now (mega-)cities in China are even larger than 10–15 years ago when the current investigated sample was growing up (although we did observe the same associations between FEAR and urbanicity in female Chinese participants when investigating the associations with urban upbringing variables and current residency; but not with the birth town variable). In addition, the urbanicity items used in accordance with previous studies may have limitations regarding the large range within the answer options. There may also be more specific critical sensitive periods during childhood development, which are obscured by focusing on the whole of the first 15 years of life. Similarly, personality development exhibits slight to moderate changes over the life span (e.g., [37,38]) and it would be interesting to see (also in light of some correlations between PETS and age) what associations between the urbanicity index and PETS occur in late adulthood. Finally, the ANPS only represents a self-report approach to one’s own PETS. Therefore, experimental measures assessing individual differences in PETS might come up with different results in the context of the urbanicity index used here. However, measurement of raw affect in adult humans is not easy given strong top-down cortical control of the subcortical regions where primal emotions reside.

## 5. Conclusions

In summary, the present study provides some first insights indicating that PETS, as an important aspect of personality, may be partly shaped by upbringing in rural or city areas influencing resilience or vulnerability for the development of psychopathologies. These effects tend to be stronger in females than in males (with the exception of PLAY in males) and might be further influenced by living in mega-cities, where effects become stronger. Finally, not all PETS are associated with urban upbringing, but rather distinct emotional systems (FEAR and SADNESS in females; PLAY in males). Clearly, this research area is complex, but our findings hopefully encourage researchers in the area of personality psychology and related psychiatric/neuroscientific fields to include such variables (different urbanicity-related variables). This will be important not only for furthering our understanding of genes by environment (urban) effects on human personality, but also to mitigate for situations where taking into account a variable environment in concert with genomic information could result in different outcomes (see also [39]). Finally, future studies should also consider using an urbanicity index, which also includes the degree of the availability of natural environments in a city. Mounting evidence suggests positive effects of natural landscapes in urban areas on mental well-being (e.g., [40]).

## Figures and Tables

**Table 1:** Primary emotional systems represent in-built tools for survival that are highly conserved across the mammalian brain (taken from Montag & Panksepp, 2017; see [21]).

Primary Emotional System (PES)	Evolutionary Function
SEEKING	The SEEKING system provides mammals with psychological “energy” (i.e., enthusiasm) to explore the environment. This is necessary to find mating partners as well as food to nourish both the brain and body.
LUST	LUST activity is of importance for attraction to the opposite sex and transfer one’s own genome (hence also of species *homo sapiens*) in terms of offspring.
CARE	Humans are social mammals. In social groups survival chances are higher. Moreover, taking CARE of one’s own offspring helps assure that the young children grow into adults and themselves can have families.
PLAY	PLAY behavior is of importance to learn social competencies and motoric skills. This aids living successfully in complex social groups as an adult.
FEAR	Without a FEAR response (along with the learning it promotes) *homo sapiens* would not have optimal abilities to escape and avoid dangerous situations and to carefully monitor the safety of their environments.
ANGER	Activity of the ANGER system is observed when mammals are in need of defending themselves (when a predator is closing in), but also in situations of frustration, when an expected reward is absent. ANGER activity is also displayed in the context of resolving territorial conflicts.
SADNESS	PANIC/SADNESS reflects separation distress and signals a situation of having lost contact with an important person or being lost in the environment. As *homo sapiens* is a social animal, separation from a caregiver or another important person triggers a distress reaction leading to distress vocalization (crying) to reunite with a partner or a parent. Ultimately, as with CARE, *homo sapiens* is stronger in groups than when alone. CARE activities can also counteract and downregulate SADNESS arousal.

**Table 2:** Internal consistencies of the ANPS in the German and the Chinese sample.

ANPS Scale	German Sample	Chinese Sample
SEEKING	0.70	0.69
FEAR	0.86	0.81
CARE	0.76	0.73
ANGER	0.85	0.80
PLAY	0.78	0.71
SADNESS	0.75	0.74
Spirituality	0.88	0.70

**Table 3:** Means (SDs) and [observed range] of all variables of interest separately for the samples from Germany and China.

ANPS Scale/Urbanicity Index	German Sample (*n* = 324)	Chinese Sample (*n* = 713)
SEEKING	40.17 (4.41)	39.33 (4.13)
	[25; 52]	[23; 53]
FEAR	36.14 (6.58)	35.80 (5.41)
	[18; 53]	[16; 56]
CARE	41.81 (5.55)	38.98 (4.99)
	[24; 54]	[18; 53]
ANGER	35.83 (6.56)	35.41 (5.59)
	[17; 56]	[17; 56]
PLAY	42.59 (5.43)	39.02 (4.62)
	[24; 56]	[23; 52]
SADNESS	34.19 (5.26)	36.46 (5.03)
	[20; 54]	[24; 52]
Spirituality	26.51 (7.01)	34.19 (3.95)
	[13; 47]	[20; 47]
Urbanicity index	25.85 (11.42)	47.87 (21.22)
	[15; 45]	[15; 75]

The possible range of scores for the SEEKING, FEAR, CARE, ANGER, PLAY, and SADNESS scales were 14–56, the possible range of points for Spirituality was 12–48 and the possible range of the urbanicity index was 15–45 in the German sample and 15–75 in the Chinese sample (see also Section 2.2).

**Table 4:** Partial correlations (controlled for age) and 95% confidence intervals between the urbanicity index and the ANPS scales.

	German Sample			Chinese Sample		
	Total (*n* = 324)	Males (*n* = 111)	Females (*n* = 213)	Total (*n* = 713)	Males (*n* = 434)	Females (*n* = 279)
SEEKING	0.01	0.03	(−)0.00	0.03	0.05	−0.01
	[−0.10; 0.10]	[−0.14; 0.20]	[−0.13; 0.13]	[−0.04; 0.10]	[−0.04; 0.13]	[−0.12; 0.10]
FEAR	−0.05	−0.03	−0.09	−0.10 **	−0.05	−0.18 **
	[−0.16; 0.05]	[−0.23; 0.17]	[−0.21; 0.04]	[−0.17; −0.02]	[−0.14; 0.05]	[−0.31; −0.05]
CARE	0.08	0.12	0.03	0.04	0.03	0.06
	[−0.02; 0.18]	[−0.08; 0.32]	[−0.10; 0.16]	[−0.03; 0.11]	[−0.07; 0.12]	[−0.06; 0.18]
ANGER	0.02	0.00	0.03	−0.06	−0.05	−0.08
	[−0.08; 0.12]	[−0.17; 0.18]	[−0.11; 0.17]	[−0.13; 0.02]	[−0.14; 0.05]	[−0.20; 0.04]
PLAY	0.01	0.01	0.02	0.10 **	0.13 **	0.06
	[−0.10; 0.11]	[−0.19; 0.20]	[−0.10; 0.14]	[0.03; 0.17]	[0.02; 0.22]	[−0.04; 0.16]
SADNESS	−0.01	(−)0.00	−0.04	−0.05	−0.01	−0.14 *
	[−0.12; 0.09]	[−0.20; 0.17]	[−0.17; 0.10]	[−0.13; 0.02]	[−0.10; 0.07]	[−0.26; −0.02]
Spirituality	0.05	0.10	0.01	0.00	0.01	−0.02
	[−0.06; 0.15]	[−0.09; 0.29]	[−0.11; 0.13]	[−0.07; 0.07]	[−0.08; 0.10]	[−0.11; 0.08]

Note. ** *p* ≤ 0.01, * *p* ≤ 0.05 (two-sided). As the urbanicity index was not normally distributed (in both samples), we also calculated Spearman correlations and checked these for significance. Neither the Spearman correlations, nor the corresponding significances did differ meaningfully from the partial correlations and significances presented in this table. Therefore, we only report statistics from parametric testing in the present results section. The confidence intervals were calculated using a bootstrapping method (1000 samples, Bias corrected and accelerated).

**Table 5:** Standardized regression coefficients for the regression analysis to explain the score in the PETS FEAR/PLAY/SADNESS.

Dependent Variable	Predictors	German Sample	Chinese Sample
FEAR	Gender	β = 0.313, t = 2.37, *p* = 0.019	β = 0.345, t = 3.73, *p* < 0.001
	Urbanicity index	β = −0.053, t = −0.57, *p* = 0.567	β = −0.034, t = −0.72, *p* = 0.475
	Gender × Urbanicity index	β = −0.073, t = −0.47, *p* = 0.637	β = −0.216, t = −2.16, *p* = 0.031
PLAY	Gender	β = 0.011, t = 0.08, *p* = 0.936	β = −0.039, t = −0.42, *p* = 0.677
	Urbanicity index	β = −0.024, t = −0.25, *p* = 0.800	β = 0.149, t = 3.08, *p* = 0.002
	Gender × Urbanicity index	β = −0.014, t = −0.09, *p* = 0.930	β = −0.071, t = −0.71, *p* = 0.480
SADNESS	Gender	β = 0.314, t = 2.37, *p* = 0.018	β = 0.411, t = 4.50, *p* < 0.001
	Urbanicity index	β = −0.017, t = −0.18, *p* = 0.858	β = 0.036, t = 0.76, *p* = 0.449
	Gender × Urbanicity index	β = −0.057, t = −0.37, *p* = 0.713	β = −0.206, t = −2.08, *p* = 0.038

Note. For these analyses gender was dummy coded (0 = male, 1 = female).

**Table 6:** Means (SDs) and partial correlations (controlled for age) and 95% confidence intervals between the urbanicity index and the ANPS Scales in the “small” Chinese sample.

	Chinese “Small” Sample			
	Correlations			
	M (SD)	Total (*n* = 290)	Males (*n* = 189)	Females (*n* = 101)
Urbanicity index	28.51 (11.44)			
SEEKING	39.18 (3.71)	0.09	0.03	0.21 *
		[−0.02; 0.20]	[−0.12; 0.18]	[0.01; 0.38]
FEAR	36.36 (5.40)	−0.02	0.07	−0.13
		[−0.13; 0.10]	[−0.08; 0.20]	[−0.33; 0.11]
CARE	38.66 (4.39)	0.03	0.10	−0.11
		[−0.08; 0.15]	[−0.03; 0.22]	[−0.29; 0.07]
ANGER	35.65 (4.99)	0.00	0.11	−0.17
		[−0.11; 0.12]	[−0.04; 0.27]	[−0.37; 0.03]
PLAY	38.46 (4.23)	0.09	0.12	0.02
		[−0.03; 0.20]	[−0.02; 0.26]	[−0.19; 0.24]
SADNESS	36.29 (4.96)	0.06	0.14	−0.07
		[−0.05; 0.17]	[−0.01; 0.30]	[−0.25; 0.13]
Spirituality	33.95 (3.71)	−0.04	−0.05	−0.02
		[−0.16; 0.08]	[−0.20; 0.10]	[−0.23; 0.18]

Note. * *p* ≤ 0.05 (two-sided). The confidence intervals were calculated using a bootstrapping method (1000 samples, Bias corrected and accelerated).
